# Development and Validation of a New HPLC Method for the Determination of Gabapentin

**Published:** 2009-03

**Authors:** Rajinder Singh Gujral, Sk Manirul Haque

**Affiliations:** *Vardhman Chemtech Ltd, Nimbua, Dera Bassi, Mohali, Punjab, India*

**Keywords:** gabapentin, potassium iodate, potassium iodide, high performance liquid chromatography

## Abstract

A simple HPLC method was developed and validated for quantitation of gabapentin in pure form. The HPLC separation was achieved on a C_18_ 5 μm Waters column (150 mm × 4.6 mm) using a mobile phase of methanol - potassium dihydrogen orthophosphate solution (20:80, v/v) containing 10% NaOH to adjust pH6.2 at a flow rate of 1.0 ml/min. The UV detector was operated at 275 nm. The method was validated for specificity, linearity, precision, accuracy, robustness and limit of quantitation. The degree of linearity of the calibration curves, the percent recoveries, limit of detection and quantitation for the HPLC method were determined. The method was found to be simple, specific, precise, accurate, and reproducible.

## INTRODUCTION

**Figure 1:**
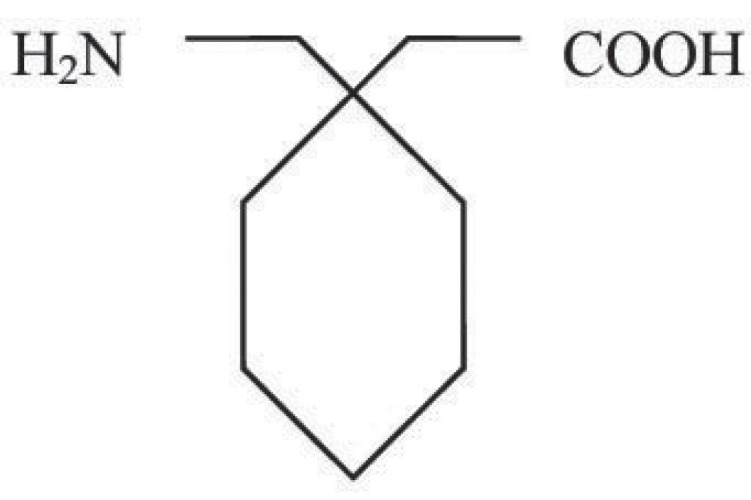
Structure of Gabapentin.

Gabapentin [1-(amino methyl)-cyclohexaneacetic acid] is a cyclic GABA [gamma - amino butyric acid] analogue (Fig. 1). Although, it is structurally related to GABA, gabapentin has no direct GABA mimetic effect. Gabapentin is originally developed for the treatment of epilepsy. It is widely used to relieve pain, especially neuropathic pain. It is well tolerated in most patients, has a relatively mild side effect profile and passes through the body unmetabolized. Its exact mechanism of action is unknown, but its therapeutic action on neuropathic pain is thought to involve voltage- gated N-type calcium ion channels. It is thought to bind to the α2∂, subunit of the voltage-dependent calcium channel in the central nervous system.

Gabapentin has been found to be effective in prevention of frequent migraine headaches (1), neuropathic pain (2) and nystagums (3) and is prescribed off- label (that is, without formed regulatory agreement) for these conditions. It has also been used in the treatment of bipolar disorder. It also acts as a mood stabilizer and the advantage of having fewer side effects than more conventional bipolar drugs such as lithium and valproic acid. It has limited usefulness in the treatment of anxiety disorders such as social anxiety and obsessive compulsive disorder, in treatment - resistant depression and insomnia (4, 5). It may be effective in reducing pain spasticity in all multiple sclerosis (6). It has also had success in treating certain instances of complex Regional Pain Syndrome. It has also been found to help patients with post - operative chronic pain (Usually caused by nerves that have served accidentally in an operation and when grown back have reconnected incorrectly). Symptoms of this include a tingling sensation near or around the area where the operation was performed.

Gabapentin was originally approved in the U.S. by the Food and Drug Administration (FDA) in 1994 for use as an adjunctive medication to control partial seizures (effective when added to other antseizures drugs).

There is a wide individual variation in the rate of clearance of these drugs and there is always a need to perform compliance testing, ascertain toxicity, and elucidate possible clinical interactions. Accordingly, the therapeutic monitoring of gabapentin is highly desirable (7).

Gabapentin is highly water soluble, with an octanol/buffer (pH7.4) log P value of -1.10, and is zwitteronic at physiological pH (pK_a_ value of 3.68 and 10.7) (8). Absolute bioavailability of gabapentin is dose dependent.

The literature reveals that numerous analytical methods have been reported for the determination of gabapentin in pharmaceutical preparations and human serum. These methods are based on Gas chromatography (GC) (9, 10), Gas chromatography - Mass spectrophotometry (GC-MS) (11), HPLC (12-17), Capillary electrophoresis (CE) (18, 19) and fluorometry (20-22). The GC methods require complex sample preparation involving double derivatization of the drugs to improve the volatility and avoid column interactions. Comparing the CE and HPLC methods, the latter is more precise, reproducible, and sensitive than the former, although the many advantages of CE, such as smaller injection volume, simplicity and wide applicability, should be taken into account. Fluorometric methods are less accurate and less specific than HPLC.

Most of the HPLC assay procedures for the determination of gabapentin are based on the same approach, involving a simple automated O - phthaldehyde (OPA) derivatization followed by HPLC separation in acidic mobile phases and fluorometric detection. Although the derivatization step is simple and rapid, the OPA - derivative was only stable for 25 min and, therefore, less suitable for routine clinical monitoring. Most of the analysis of gabapentin was depend on derivatization with other reagent. In these cases, the derivatization condition was time consuming and the stability of the reaction products depends on experimental conditions such as pH, temperature and reaction time.

This paper describes a sensitive, fast, simple and economical method for the determination of gabapentin in pure form. The method is based on the reaction of carboxylic acid group of gabapentin with a mixture of potassium iodate and iodide.

## EXPERIMENTAL

### Materials

- Gabapentin (Vardhman Chemtech Ltd, Mohali, Punjab, India) used as an internal standard (0.9923 mg/mg) [Standardized with USP Gabapentin (0.999 mg/mg) Lot No GOEOO5.
- Potassium iodate (KIO_3_) was purchased from RFCL Limited (New Delhi, India).
- Potassium iodide (KI) was purchased from RFCL Limited (New Delhi, India).
- Methanol was HPLC grade and purchased from Qualigens fine Chemicals, Mumbai, India.
- Potassium dihydrogen orthophosphate (KH_2_PO_4_) was purchased from Qualigens fine Chemicals, Mumbai, India.
- All other chemicals were of analytical grade and used without any further purification.

### Instrumentation

- The HPLC used was model LC-2010 CHT, Shimadzu, Kyoto, Japan with pump model 2 LC-10 ADvp, Autosampler model SIL-10 ADvp, Column oven model CTO-10 A (C) vp.
- The detector was a UV detector model SPD-10 A (V) vp. The system was driven by a HP-5502.
- The 2487 Waters HPLC (USA) with 515 pumps was also used. The system was driven by Sync Master 794 MG.
- Digital pH- meter, G-200/A, HPG systems (Mumbai, India)
- The data processing system were run with Breeze software for 2487 Waters HPLC and LC solution for LC-2010 CHT (Shimadzu).
- The HPLC column used was a Waters C_18_ 5 μm column (150 mm × 4.6 mm). The mobile phase filtration unit was Ultipor^®^ N_66_^®^ Nylon 6, 6 membrane (Pall Life Sciences, Mumbai, India), Lot No-06-07-ID 000784.

### Chromatographic system and conditions

HPLC method was performed using a Waters 515 HPLC pump, Waters 2487 Dual λ absorbance detector (Waters, USA). Separation was operated on a C_18_ 5 μm Waters column (150 mm × 4.6 mm). The mobile phase consisted of methanol - potassium dihydrogen orthophosphate (20:80, v/v) at a flow rate of 1.0 ml/min. Potassium dihydrogen orthophosphate solution was prepared by dissolving 3.811 gm KH_2_PO_4_ in 800 ml double distilled water. Final pH of the mobile phase was adjusted to 6.2 by 10 % NaOH.

## METHODS

### Procedure for determination of gabapentin

**Figure 2:**
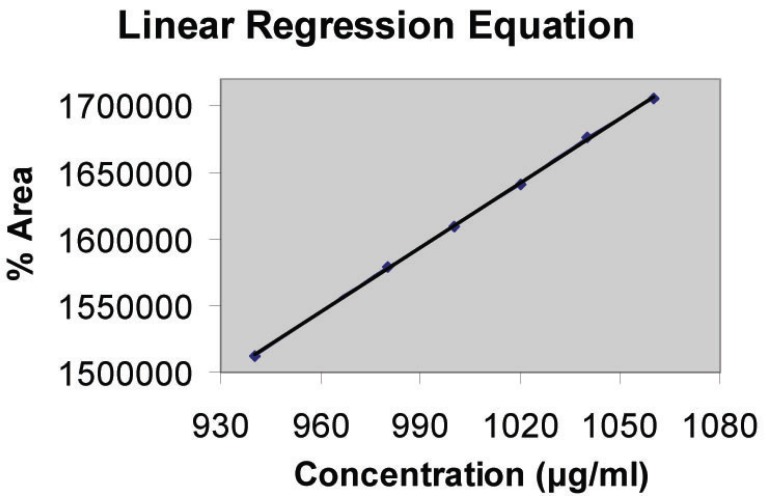
Linearity of the proposed method.

Aliquots of stock solution (20 mg/ml) were pipetted into a series of 10 ml volumetric. To each flask, 0.8 ml 5 × 10^-1^ M KI and 3.2 ml 3 × 10^-1^ M KIO_3_ were added and diluted to volume with distilled water. The reaction was allowed to proceed at room temperature. The calibration curve was constructed by plotting peak area against the initial concentration of gabapentin. The linearity range or Beer’s range follows in the range between 940 to 1060 μg/ml (Fig. 2). The content of gabapentin was calculated either from the calibration curve or corresponding regression equation and found that the peak area is stable for at least ten days at room temperature.

### Procedure for reference method (23)

Aliquots of stock solution (14 mg/ml) were transferred into a set of 10 ml volumetric flasks and volumes were completed to the mark with diluents (dissolve 2.32 gm monobasic ammonium phosphate in 1000 ml of water. Adjust with phosphoric acid to a pH of 2.0) to produce solutions in the concentration range of 1000-10000 μg/ml. Calibrations were constructed by plotting peak area against the final concentration gabapentin.

For reference method the separation was achieved by using 4.6 mm × 25 cm column that contains packing L1. The isocratic mobile phase pumped at a flow - rate of 1.0 ml/min consisted of acetonitrile - buffer solution (Dissolve 0.58 gm of monobasic ammonium phosphate and 1.83 gm of sodium perchlorate in 1000 ml of water. Adjust with perchloric acid to a pH of 1.8).

### Analysis of Commercial dosage form

To determine the content of gabapentin in tablet formulation (label claim: 800 mg), the contents of 5 tablets were weighed and finely powdered. A portion of the powder equivalent to 2000 mg gabapentin was stirred with 100 ml distilled water and let stand for 10 min. The residue filtered with Ultipor^®^ N_66_^®^ Nylon 6, 6 membrane (Pall Life Sciences, Mumbai, India), Lot No-06-07-ID 000784. This solution was subjected to the proposed procedure for the determination of gabapentin.

### Procedure for the determination of gabapentin in patients’ specimens

Aliquot volumes of patients’ specimens samples were transferred into small separating funnel. 5 ml of carbonate buffer pH - 9.4 (prepared by dissolving 26.5 gm sodium carbonate and 21.0 gm sodium bicarbonate in 500 ml distilled water) was added and solution was mixed well. The solution was then extracted with 3 × 5 ml of diethyl ether. The ether extract was collected and evaporated. The residue was dissolved in 5 ml of distilled water and above general procedure was then followed. The nominal content of gabapentin was determined from the corresponding regression equation.

## RESULTS AND DISCUSION

### Reaction with a mixture of iodide and iodate

It has been reported in the literature (24) that iodine is formed as a result of the interaction of a mixture of iodide and iodate with inorganic or organic acid in accordance with the equation:

${5I}^{-}+{\mathrm{IO}}_{3}^{-}+{6H}^{+}\to {3H}_{2}O+{3I}_{2}$

**Figure 3:**
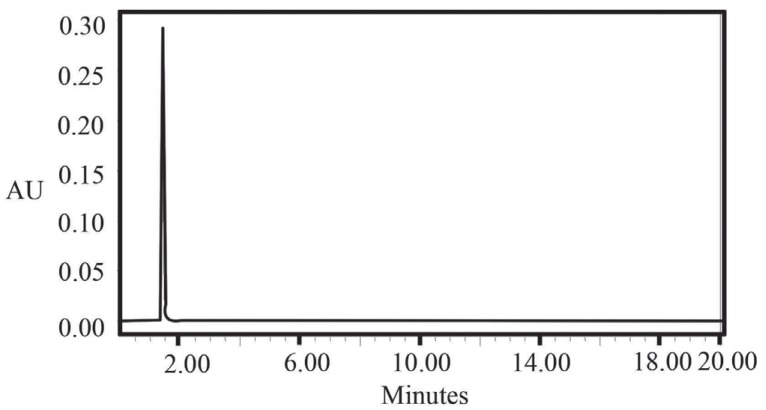
HPLC Chromatograms of Gabapentin (2 mg/ml); keeping constant of 0.8 ml 5 × 10^-1^ M KI and 3.2 ml 3 × 10^-1^ M KIO_3_.

**Figure 4:**
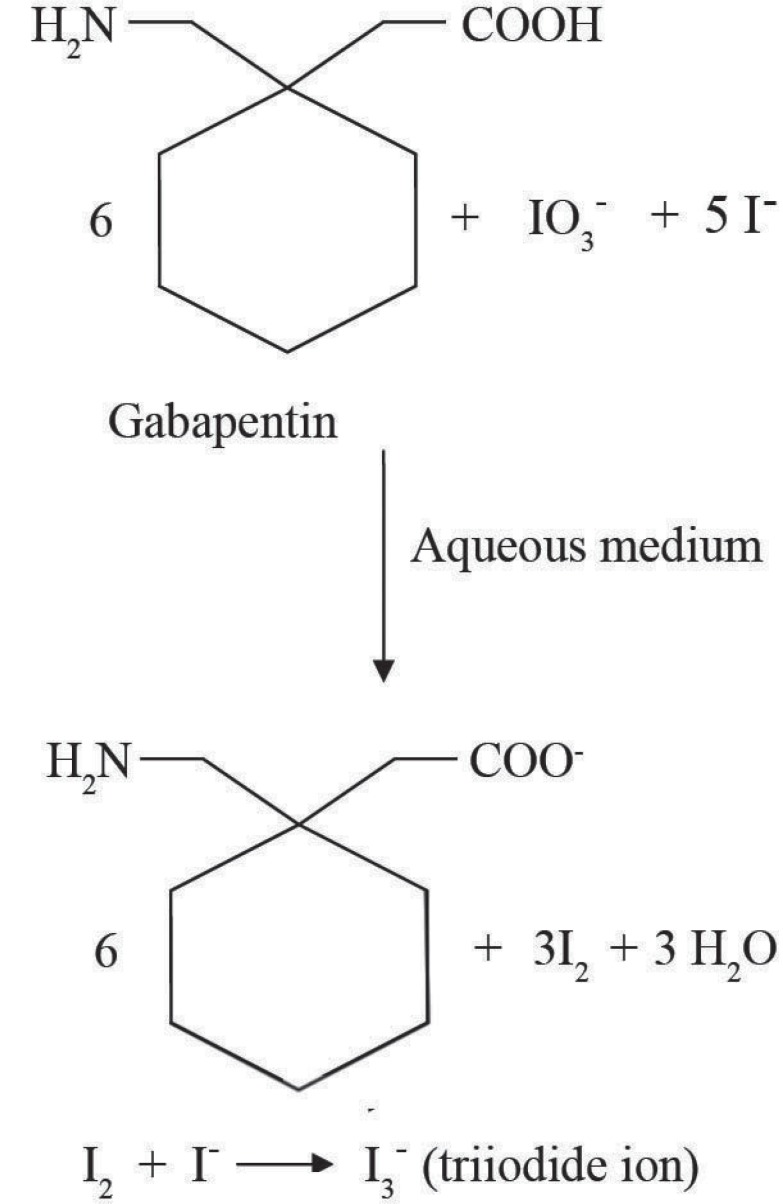


In aqueous medium, the iodide ions react with the liberated iodine to yield triiodide ion (I_2_ + I^-^ → I_3_^-^) which detected in UV detector at 275 nm (Fig. 3). We thought that this reaction would be helpful for developing a HPLC method for determination of gabapentin as it contains -COOH group in its moiety. Keeping this in mind, a mixture of potassium iodide and iodate was allowed to react with gabapentin which yielded iodine. Then the liberated iodine reacted with the excess of iodide ion resulting in the formation of triiodide ion (Fig. 4).

### Optimization of reaction conditions

The different parameters affecting the development process were extensively studied to determine the optimum conditions for the assay procedures. The optimum values of the variables were maintained throughout the determination process.

**Figure 5:**
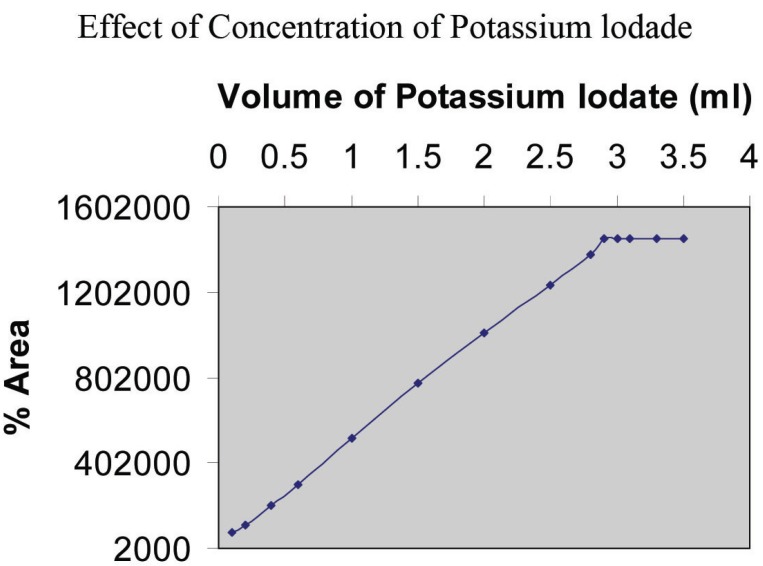
Effect of the volume of KIO_3_ (3.0 × 10^-3^ M); keeping constant 1 ml of 2 mg/ml gabapentin and KI (0.8 ml of 5.0 × 10^-1^M).

**Effect of the concentration of potassium iodate:** The effect of the volume of 3 × 10^-1^ M potassium iodate on the peak area of the product was studied in the range of 0.1-3.5 ml. The peak area increases with the increase in the volume of potassium iodate and became constant at 2.9 ml. Further addition of KIO_3_ does not change in the peak area and therefore, 3.2 ml of 3 × 10 ^-1^ M KIO_3_ was chosen as an optimum value (Fig. 5).

**Figure 6:**
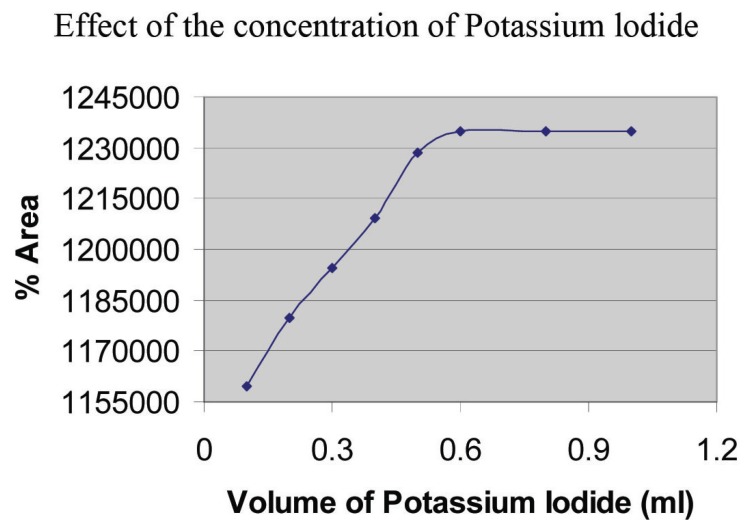
Effect of the volume of KI (5.0 × 10^-1^ M); keeping constant 1 ml of 2 mg/ml gabapentin and KIO_3_ (2.5 ml of 3.0 × 10^-1^M).

**Effect of the concentration of potassium iodide:** The effect of the volume 5 × 10^-1^ M potassium iodide on the peak area of the product was studied in the range of 0.1 - 1.0 ml, keeping the constant concentrations of gabapentin (2 mg/ml) and KIO_3_ (7.5 × 10^-2^ M). The maximum peak area was obtained with 0.6 ml; further addition caused no change on the peak area. Thus, 0.8 ml of 5 × 10^-1^ M potassium iodide (Fig. 6) was used throughout the experiment.

### Validation of proposed method

Validation of analytical procedures is a vital aspect not just for regulatory purposes, but also for their efficient and reliable long - term application. The ICH guidelines achieved a great deal in harmonizing the definitions of required validation parameters, their calculation and interpretation. It is the responsibility of the analyst to identify parameters which are relevant to the performance of the given analytical procedure as well as to design proper validation protocols including acceptance criteria and to perform an appropriate evaluation. The international conference on the Harmonization of the Technical Requirements for Registration of Pharmaceuticals for Human Use (ICH) has harmonized the requirements in two guidelines (25, 26). The first one summarizes and defines the validation characteristics needed for various types of test procedures, the second one extends the previous test to include the experimental data required and some statistical interpretation. These guidelines serve as a basis worldwide both for regulatory authorities and industry and bring the importance of a proper validation to the attention of all those involved in the process of submission. Nowadays, the validation characteristics needed for the various test procedures and their general requirements are well understood. The essential question to be answered is on the suitability of the calibration mode to be used in the test procedure. It should be noted that in most cases only a qualitative statement is needed.

Under the optimum experimental conditions, the peak area - concentration plot for the proposed method was found to be rectilinear over the range of 940-1060 μg/ml (Fig. 2). The suitability of the reaction products was estimated in the reaction mixture, prior to injection into the HPLC system, it was found that the peak area is stable for at least ten days at room temperature.

The specificity of the method was investigated by observing any interference encountered from the excipients of the tablets. It is shown that these additives do not interfere with the propose method. The within day precision assays were carried out through replicate analysis (n=5) of gabapentin corresponding to 940, 980 and 1040 μg/ml. The interday precision was also evaluated through replicate analysis of the pure drug samples for five consecutive days at the same concentration levels as used in within day precision. The results of these assays are reported in Table 1. As can be seen from Table 1 that Recovery values for intraday and interday precision were in the range of 99.960 to 100.038 % and RSD values for intraday and interday precision were in the range of 0.031 to 0.115 %. The accuracy was ascertained by recovery studies using the standard addition method. The results are summarized in Table 2. As can be seen from Table 2 that the recovery and RSD values for the proposed method were in the range of 99.961 to 100.009 % and 0.034 to 0.156 %. There is no interference from excipients present in tablet formulation. The proposed method was also validated for low purity of gabapentin and compared with the reference method (23). The precision results are satisfactory (Table 3).

**Table 1:** Intra and inter day precision of the HPLC method for determination of Gabapentin

Parameters	Concentration (μg/ml)	Mean	SD	Recovery %	RSD %	SAE	CL

Intraday	940.00	939.601	1.076	99.960	0.115	0.481	1.336
	980.00	980.381	0.554	100.038	0.056	0.248	0.688
	1040.00	1040.125	0.357	100.012	0.034	0.160	0.443
Interday	940.00	939.769	0.605	99.975	0.064	0.270	0.750
	980.00	979.986	0.299	99.998	0.031	0.134	0.372
	1040.00	1039.995	0.573	99.995	0.055	0.256	0.711

**Table 2:** Summary of data for the determination of gabapentin in pharmaceutical formulation by standard addition method

Tablet	Amount (μg/ml)			Recovery %	RSD %	SAE	CL
	Taken	Added	Found ± SD				

Gabacom	900	50.0	949.629 ± 1.480	99.961	0.156	0.662	1.837
	900	100.0	999.947 ± 0.338	99.995	0.034	0.151	0.419
	900	150.0	1050.099 ± 0.450	100.009	0.043	0.201	0.559

**Table 3:** Assay results of gabapentin in commercial tablet using the proposed and reference method (23)

Method	Gabapentin %	Mean	Recovery %	RSD %	SAE	CL

Proposed	99.90	1039.995	99.999	0.055	0.256	0.711
	94.23	977.682	94.008	0.074	0.324	0.899
	88.75	923.026	88.752	0.703	0.903	2.501
Reference	99.90	1040.018	99.999	0.034	0.161	0.447
	94.23	981.791	94.403	0.046	0.202	0.562
	88.75	923.888	88.835	0.149	0.613	1.702

The performance of the proposed method was studied with other existing HPLC method (27). In case of the proposed method and reported method, the accuracy of the reported method is somewhat poor with relatively higher RSD value and the drawback is that it requires dissolution samples and dissolution medium for the analysis. Therefore, the proposed method was found to be simple and can compete with other existing HPLC method for the determination of gabapentin.

**Figure 7:**
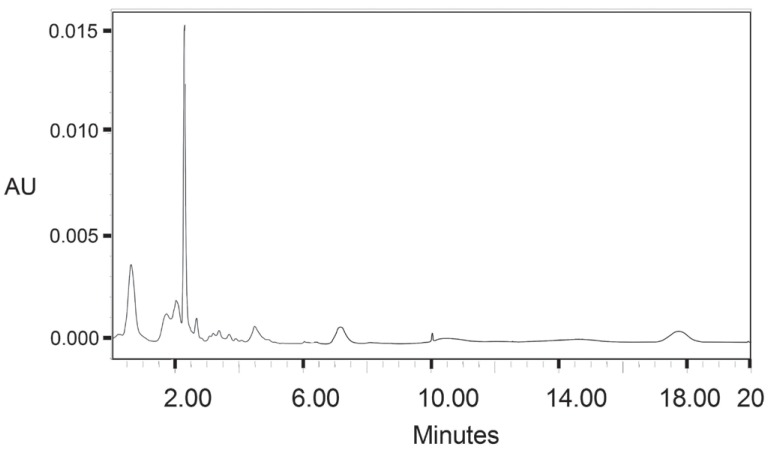
HPLC chromatograms of serum samples.

The proposed method was further extended to the *in vitro* determination of gabapentin in human plasma samples in the proposed linearity range and also in low concentrated plasma samples. The results of analysis of plasma samples are summarized in Table 4 and 5. These results are satisfactorily accurate and precise. The contaminating chemicals with COOH structure in the serum was used as a blank (Fig. 7) in the HPLC method which showed at retention times 2.29 but the serum with gabapentin showed retention time 1.39 (Fig. 3). Therefore the proposed method was found to be accurate for *in vitro* determination of gabapentin in human serum and plasma samples.

**Table 4:** Application of the proposed HPLC method to the determination of gabapentin in plasma samples

Amount added (μg/ml)	Amount found (μg/ml)	Recovery (%)

940.0	945.33	100.56
960.0	947.59	98.71
980.0	968.55	97.81
1000.0	979.32	97.93
1020.0	1008.02	98.83
1040.0	1023.12	98.38
X		98.70
RSD		1.01

**Table 5:** Application of the proposed HPLC method to the determination of gabapentin in low concentrated plasma samples

Amount added (μg/ml)	Amount found (μg/ml)	Recovery (%)

1.0	1.01	101.00
3.0	2.92	97.33
5.0	4.87	97.40
10.0	9.82	98.20
20.0	19.62	98.10
30.0	29.50	98.33
40.0	39.21	98.03
50.0	50.10	100.20
60.0	58.90	98.16
X		98.53
RSD		1.26

## CONCLUSION

The proposed method does not require any laborious clean up procedure before measurement. In addition, the method has wider linear dynamic range with good accuracy and precision. The methods show no interference from common excipients. The statistical data and recovery data reveal the good accuracy and precision of the proposed method. Therefore, it is concluded that the proposed method is simple, sensitive and rapid for the determination of gabapentin in pure and human plasma samples.
